# Use of the oxygen reserve index/FiO_2_ as a non-invasive index to estimate venous admixture in anesthetized dogs

**DOI:** 10.3389/fvets.2024.1495543

**Published:** 2025-01-06

**Authors:** Luca Bellini, Jill K. Maney, Francesca Zanusso, Brighton T. Dzikiti

**Affiliations:** ^1^Department of Animal Medicine, Production and Health, University of Padova, Padova, Italy; ^2^Department of Clinical Sciences, Ross University School of Veterinary Medicine, Basseterre, Saint Kitts and Nevis

**Keywords:** F-shunt, dogs, oxygen reserve index, anesthesia, blood gas analysis

## Abstract

The oxygen reserve index (ORi) is a novel, non-invasive parameter that estimates arterial oxygen partial pressure (PaO_2_) during hyperoxia when the fraction of inspired oxygen (FiO_2_) is elevated. This study aimed to assess the utility of the ORi/FiO_2_ ratio as an index for quantifying F-shunt, serving as an estimate of venous admixture. Anesthetic records were reviewed from 44 dogs undergoing general anesthesia and requiring arterial catheterization. ORi was measured via a CO-oximeter using a probe on the tongue. Paired measurements of PaO_2_, obtained by blood gas analysis, and ORi were taken at various FiO_2_ levels to achieve an ORi between 0 and 1. Venous admixture was quantified by F-shunt. Spearman’s correlation coefficient assessed the relationship between ORi/FiO_2_ and F-shunt. Youden’s index identified the optimal cut-off point to predict a physiological F-shunt (≤ 10%). A total of 77 paired observations were collected, revealing a moderate negative correlation between F-shunt and ORi/FiO_2_ (rho = −0.59, *p* < 0.001). An ORi/FiO_2_ cut-off of 1.2 demonstrated 80% sensitivity for identifying dogs with an F-shunt ≤10%, with a ROC curve area above 80%. However, the index was less effective at distinguishing dogs with higher shunt fractions. The ORi/FiO_2_ index identifies dogs with low F-shunt during anesthesia with strong sensitivity and predictive accuracy, potentially ruling out the occurrence of ventilation-perfusion inequality. However, it cannot replace blood gas analysis for quantifying venous admixture.

## 1 Introduction

To prevent complications under anesthesia, an acceptable level of arterial oxygen content is essential. This is accomplished through adequate ventilation and gas exchange in the lungs, ensuring an appropriate level of partial pressure of oxygen in the arterial blood (PaO_2_). Muscle relaxation and hemodynamic changes caused by volatile or injectable anesthetics can reduce lung volume and impair ventilation, leading to atelectasis, intrapulmonary shunting, and ventilation-perfusion mismatch (1). The severity of these processes can be estimated using the venous admixture, which measures the extent to which poorly oxygenated or deoxygenated blood mixes with fully oxygenated blood from the lungs (1). F-shunt quantifies venous admixture using variables obtained from the arterial blood gas analysis, the patient hemoglobin concentration and inspired fraction of oxygen (FiO_2_). This parameter showed a high correlation with venous admixture measured invasively in sheep and horses (2, 3), and it has also been used in dogs (4–6). In healthy, anesthetized canine patients, the fraction of pulmonary blood shunting to the left side of the heart should not exceed 10% (1). However, under general anesthesia, conditions such as obesity or high FiO_2_ can increase this fraction due to a decrease in thoracic volume or absorption atelectasis, respectively (7, 8).

In human patients, the PaO_2_/FiO_2_ ratio (P/F) serves as a surrogate for venous admixture and, although it has some limitations, in cases of respiratory distress syndrome it correlates well with the severity of anatomical abnormalities observed by CT scanning (9). Similarly, in dogs, an increase in non-ventilated areas of the lung identified by CT scan is significantly associated with a decrease in the P/F ratio (8). Despite this, a limitation of this ratio is that it requires arterial sampling via an arterial catheter or puncture, both of which carry potential complications, such as hematoma or bacterial contamination, and provide intermittent, delayed data. Consequently, non-invasive alternatives have been investigated. Peripheral pulse oximetry (SpO₂) offers a non-invasive estimate of oxygenation, as it linearly correlates with PaO₂ values between 80 and 100 mmHg (10). The ratio of SpO_2_/FiO_2_ (S/F) demonstrated diagnostic validity as an estimate for P/F in patients with hypoxia or respiratory disfunction (11). In dogs, the ratio of hemoglobin saturation to FiO₂, assessed using arterial oxygen saturation (SaO₂) and SpO₂, shows a strong to moderate correlation with the P/F ratio when saturation is below 97% in patients breathing room air, in spontaneous or controlled mechanical ventilation (10, 12). Although the S/F ratio is easy to calculate, its clinical significance in estimating venous admixture in dogs remains unclear. Additionally, it becomes less relevant during sedation or anesthesia when FiO₂ exceeds 0.3, resulting in SpO₂ values between 97 and 100% regardless of PaO₂ (13, 14).

The oxygen reserve index (ORi) is a dimensionless parameter that estimates the PaO_2_ during mild hyperoxaemia, ranging from 0.0 (indicating PaO_2_ < 100 mmHg) to 1.0 (indicating PaO_2_ > 200 mmHg). The ORi is measured using a multi-wavelength pulse CO-oximeter, which identifies and compares the absorption of light at different wavelength due to oxygen-bound hemoglobin in both arterial and venous blood. In veterinary medicine, the ability of ORi to estimate PaO_2_ was investigated in anesthetized donkeys, revealing only a weak correlation (15). In contrast to that study, a stronger correlation up to PaO_2_ values of 240 mmHg was demonstrated in anesthetized dogs, which is slightly above the theoretical value of 200 mmHg (13, 16). Besides the ORi, the pulse CO-oximeter measures peripheral perfusion at the measurement site using the perfusion index (PI). However, the diagnostic performance of ORi in accurately detecting PaO_2_ showed high uncertainty when PI exceeded 2. The ORi was applied to monitor the response to oxygen administration in sedated dogs or to identify impending decreases in arterial oxygen content before any clinically relevant changes in SpO_2_ (14, 17). Because ORi overcomes the clinical limitations of traditional SpO_2_ in the presence of elevated PaO_2_, this new index could be used to non-invasively and continuously quantify venous admixture as ORi/FiO_2_ (O/F) in anaesthetized patients or those inhaling an oxygen-enriched gas mixture.

The primary aim of this study was to assess whether the O/F ratio could estimate venous admixture, expressed as F-shunt, in dogs undergoing general anesthesia. Additionally, a secondary aim was to identify an optimal cut-off value for O/F that predicts an F-shunt of ≤10% during anesthesia.

## 2 Materials and methods

### 2.1 Animals and study design

The Animal-welfare committee of the University of Padova approved the study protocol (OPBA Authorization 55/2021). The anesthetic records of 44 adult dogs of various breeds were analyzed. This included 37 records obtained from a previous study (13) and 7 newly collected records from additional dogs to increase the available data. The included animals, comprising 20 males and 24 females, were categorized as ASA physical status classifications 1 or 2, with all dogs being older than 9 months and weighing more than 10 kg. The body condition score (BCS) was assessed using a 9-point scale, as previously described (18). The dogs were admitted to the University of Padova Veterinary Teaching Hospital and underwent soft tissue surgeries or diagnostic imaging procedures under general anesthesia. As part of the inclusion criteria, the study included dogs with normal complete blood test results conducted within the previous three days. Additionally, animals were enrolled if anesthesia required the insertion of an arterial catheter. An arterial line was inserted in situations where non-invasive systemic blood pressure methods were not applicable due to equipment interference, such as during an MRI, or when intraoperative blood gas analysis or significant blood loss was anticipated. Dogs were excluded if they were scheduled for thoracic surgery, had a clinical history of respiratory signs such as coughing, labored breathing, or nasal discharge within the past three weeks, or had a confirmed or suspected diagnosis of intracranial pathologies.

### 2.2 Anesthetic protocol and monitoring

Food was withheld for 8 h before anesthesia. The premedication protocol was chosen based on the specific procedure they were scheduled for. Dogs undergoing diagnostic procedures (CT scan and MRI) received butorphanol (Torphedine 10 mg/mL, Dechra Veterinary Products S.r.l., Italy) 0.1–0.2 mg/kg alone or with dexmedetomidine 2–4 μg/kg (Dexdomitor 0.5 mg/mL, Vétoquinol Italia S.r.l., Italy), while dogs scheduled for surgical procedures received methadone 0.2 mg/kg (Semfortan 10 mg/mL, Eurovet Animal Health B.V., Italy) instead of butorphanol for preemptive analgesia. All agents were administered intramuscularly before placing an intravenous catheter. General anesthesia was induced intravenously with propofol (PropoVet 10 mg/mL, Zoetis, Italy) until the laryngeal reflex was suppressed to allow safe orotracheal intubation. Immediately after, the dogs were connected to a ventilator set to pressure-controlled mode, with peak inspiratory pressures ranging from 10 to 12 cmH_2_O. The ventilator generated an intrinsic positive end-expiratory pressure (PEEP) of 2 to 3 cmH_2_O. The end-tidal carbon dioxide pressure was maintained between 35 and 45 mmHg by adjusting the respiratory rate. Anesthesia was maintained with sevoflurane or isoflurane vaporized in a mixture of oxygen and air, to obtain a FiO_2_ between 0.22 and 0.50, at the discretion of the attending anaesthetist. During anesthesia, lactated Ringer’s solution (B. Braun, Italy) was infused IV at a rate of 3–5 mL/kg/h.

An arterial catheter (22G, Denta Med, Italy) to facilitate blood pressure measurement and blood sampling was aseptically inserted into the dorsal pedal or palmar artery after anesthetic induction. Anesthesia monitoring included direct systemic arterial blood pressure, end-tidal carbon dioxide, and FiO_2_, all displayed on a multi-parameter monitor (Datex S/5, GE Healthcare, Finland), which also continuously displayed the electrocardiogram trace and temperature for surgical cases. The plethysmogram and SpO_2_ were continuously recorded by a multi-wave pulse CO-oximeter (Rad-97, Masimo Corp., USA). If SpO_2_ fell below 97%, the FiO_2_ was adjusted to achieve PaO_2_ values above 100 mmHg, as determined by blood gas analysis. Hypotension (herein defined as mean blood pressure < 65 mmHg) was managed by reducing the volatile agent or administering a 5 mL/kg crystalloid bolus over 10 min. Inotropes or vasopressors were administered if necessary.

### 2.3 Data collection and blood gas analysis

The ORi and the perfusion index (PI) were measured utilizing a multi-wavelength pulse CO-oximeter (Rad-97, Masimo Corp., CA, USA), employing an adhesive sensor probe (RD Rainbow Lite SET-1 Neo, Masimo Corp., CA, USA). The emitter and detector portions of the sensor were applied to the edges of the tongue, with the adhesive bandage of the probe wrapped around it to secure the sensor, and the tongue was folded to ensure proper contact. The probe was positioned on the tongue and then connected to the instrument. Measurements were taken throughout the anesthetic maintenance phase, without specific time intervals for data collection. Consecutive measurements were conducted with a minimum difference in FiO_2_ of 0.05. Data collection commenced after a 10-min stabilization period following any adjustment in inspired oxygen concentration. ORi readings were recorded when the heart rate correlated with the pulse rate recorded by the CO-oximeter, and the pulse wave remained stable for at least 2 min. No studies have evaluated the effect of treatments for intraoperative hypotension on the accuracy of ORi in estimating PaO_2_ in animals or human patients; therefore, no data were collected from dogs until 30 min had elapsed from the end of treatment of hypotensive episodes.

The measurement of PaO_2_ was conducted using a blood gas analyzer (EDAN i15, EDAN, China) with a multi-parameter cartridge (Test Cartridge BG10, EDAN, China). Two milliliters of blood were discarded initially to prevent contamination before drawing the sample through a pre-heparinized syringe (Pulset, Westmed Inc., AZ, USA) over two to three breaths. Blood samples were withdrawn from a three-way stopcock directly connected to the arterial catheter. After blood collection, the catheter was flushed with 1 mL of heparinized solution (10 IU/mL).

The PaO_2_ value measured from the blood sample, the ORi and FiO_2_ values measured at the time of sampling, as well as the partial pressure of carbon dioxide (PaCO_2_), SaO_2_, and hematocrit obtained from the blood gas analysis, were recorded in a datasheet.

### 2.4 Raw data extraction and calculations

Raw data from 44 dogs were extracted and 116 measurements of ORi paired with the corresponding PaO_2_ were identified. Exclusion criteria were an ORi of 0, where the O/F ratio cannot be determined, or 1, where hyperoxemia ranges from 200–240 mmHg to 500 mmHg. Moreover, paired measurements were excluded if the associated PI was >2, as a recent study demonstrated that the accuracy of ORi in estimating PaO_2_ is lower for PI >2 (19).

F-shunt were calculated using as previously reported formula (6, 7):


$$\frac{\left(C\overset{´}{c}o_{2}-Cao_{2}\right)}{\left(C\overset{´}{c}o_{2}-Cao_{2}+3.5\;\mathrm{mL}/\mathrm{dL}\right)}\times 100$$

$C\overset{´}{c}o_{2}$ represents the pulmonary end-capillary oxygen content, ${Cao}_{2}$ denotes the arterial oxygen content, and 3.5 mL/dL is a constant that approximates the difference between arterial and mixed venous oxygen content. The values for $C\overset{´}{c}o_{2}$ and ${Cao}_{2}$ were determined as follows:


$$C\overset{´}{c}o_{2}=Hb\times 1.31\times S\overset{´}{c}o_{2}+0.003\times P\overset{´}{c}o_{2}$$

$$Cao_{2}=Hb\times 1.31\times Sao_{2}+0.003\times \mathrm{Pa}o_{2}$$

Hemoglobin concentration (Hb) was measured using a blood gas analyzer, with 1.31 mL/g representing the oxygen-carrying capacity of hemoglobin, $S\overset{´}{c}o_{2}$ and ${Sao}_{2}$ indicating the pulmonary end-capillary and arterial oxygen saturation, respectively and 0.003 being the solubility coefficient of oxygen in dog plasma.

The pulmonary end-capillary partial pressure of oxygen, $P\overset{´}{c}o_{2}\text{,}$ was considered equivalent to the alveolar partial pressure of oxygen and was calculated using the following formula:


$$P\overset{´}{c}o_{2}=\left(Fio_{2}\times \left[\mathrm{PB}-PH_{2}O\right]\right)-\left(Paco_{2}/0.8\right)$$

PB was the atmospheric pressure and as $P\overset{´}{c}o_{2}$ was consistently above 100 mmHg, $S\overset{´}{c}o_{2}$ was assumed to be 100% (i.e., 1).

### 2.5 Statistics

Statistical analysis was conducted with RStudio (RStudio, PBC, Boston, MA, US) as the interface for R (The R Foundation for Statistical Computing, Austria). The Shapiro–Wilk test assessed the normal distribution of continuous parameters. Normally distributed data are presented as mean ± standard deviation, while non-normally distributed values are presented as median (minimum-maximum). Correlation between P/F and O/F, and O/F with F-shunt, was determined by Pearson’s or Spearman’s correlation coefficient, based on whether the data were normally distributed or not, respectively.

Youden’s index identified the optimal cut-off value of O/F to predict an F-shunt of ≤10%. The test provided the area under the receiver operating characteristic curve (AUC), which was calculated as a measure of accuracy for discriminating between dogs with an F-shunt ≤10%. AUC values were interpreted as follows: excellent (AUC ≥ 0.90), good (0.90 > AUC ≥ 0.80), fair (0.80 > AUC ≥ 0.70), and poor (0.70 > AUC ≥ 0.60) discrimination, with 0.60 > AUC ≥ 0.50 indicating discrimination no better than chance (20). Statistical significance was set at *p* < 0.05.

## 3 Results

Dogs weighed 27 ± 8 kg, with a mean age of 95 ± 41 months, and a median BCS of 6 (range 3–9). Four dogs experienced hypotension and were treated with an intravenous crystalloid bolus (5 mL/kg over 10–15 min) at least 30 min before arterial blood sampling. None required inotropes or vasopressors. All animals completed the study without significant complications during the intra- or postoperative period.

Records from the 44 dogs produced 116 measurements. Of these, 15 were excluded because the ORi value measured was 0 or 1, and 24 were excluded because the PI was >2 or missing. The analysis was conducted on the remaining 77 measurements. Of the original number of patients enrolled, all measurements from six dogs were excluded for various reasons. One dog had all its ORi values as 1, another had both missing PI values and an ORi of 1, and four dogs had consistent PI values greater than 2. All 77 measurements were obtained while the animals were breathing an FiO_2_ between 0.22 and 0.66, with two measurements exceeding 0.5, the upper limit of the target range for this study. The hemoglobin plasma concentration recorded from the 77 measurements was 11.7 ± 3.0 g/dL, and the median PI was 1.2 (range 0.2–2.0).

**Figure 1:**
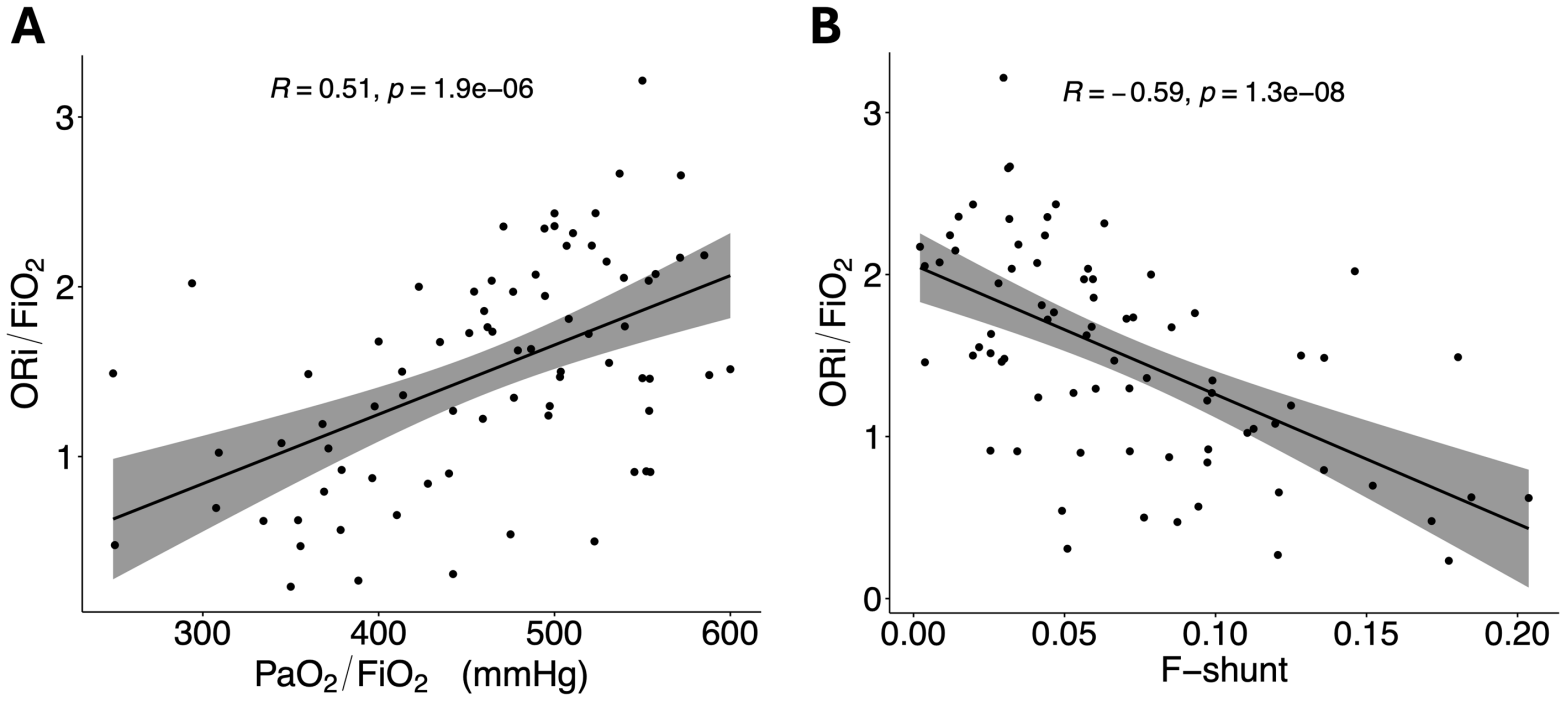
**(A)** Scatterplot showing the PaO_2_/FiO_2_ ratio. **(B)** Scatterplot showing the ORi/FiO_2_ ratio. A regression line has been added, with the grey area representing the 95% confidence intervals. ORi, oxygen reserve index; PaO_2_, partial pressure of oxygen; FiO_2_, fraction inspired of oxygen; R, Spearman’s correlation coefficient.

Mean ORi was 0.49 ± 0.23, and PaO_2_ was 147 ± 30 mmHg, while the median FiO_2_ was 0.3 (range 0.22–0.66). The median P/F ratio was 473 (range, 249–600) mmHg, and the median O/F ratio was 1.50 (range, 0.23–3.21). SpO_2_ remained above 98% in all dogs. The Spearman’s correlation coefficient between the P/F and O/F ratios was 0.51 (*p* < 0.001; Figure 1A), while between F-shunt and O/F it was −0.59 (p < 0.001; Figure 1B). Sixteen (21%) dogs had an F-shunt above 10%.

The Youden index identified an O/F cut-off value of 1.2, which provided the highest sensitivity and specificity in discriminating dogs with an F-shunt below 10%, demonstrating good diagnostic performance with an AUROC (area under the ROC curve) greater than 80 (Table 1). O/F values below this cut-off indicate an F-shunt ≥0.1. Five out of 61 (8%) dogs with an O/F ratio above 1.2 were classified as having an F-shunt greater than 10%.

**Table 1:** Area under the curve (AUC) obtained from the receiver operating characteristic curve analysis to identify the cut-off value for the oxygen reserve index (ORi) that predicts an F-shunt ≤10% in anesthetized dogs.

Estimate	Value (95% CI)
Best threshold	1.2
AUC	0.81 (0.69–0.92)
Youden Index	0.55
Sensitivity	0.80 (0.68–0.89)
Specificity	0.75 (0.48–0.92)
Positive predictive value	0.92 (0.79–0.96)
Negative predictive value	0.50 (0.34–0.81)

The cut-off value identified by the Youden index is reported along with the associated sensitivity, specificity, positive predictive value, and negative predictive value, all expressed as percentages. The 95% confidence interval (CI) is provided in brackets.

## 4 Discussion

In this study, the O/F ratio exhibited a negative correlation with the F-shunt, which estimates venous admixture. Although the observed correlation was moderate and the data tended to be dispersed, an O/F ratio above 1.2 was identified as the threshold with the highest sensitivity for predicting F-shunt values below 10%.The O/F ratio might help overcome the limitations of using hemoglobin oxygen saturation to estimate the P/F ratio as an alternative to venous admixture, particularly in cases requiring oxygen supplementation, where saturation levels remained above 97% despite increases in FiO_2_ (12). In the current study, the FiO_2_ was targeted between 0.22 and 0.5, an acceptable range administered to sedated or anesthetized animals or during the recovery period, but it may interfere with the accuracy of oxygen estimation using SpO_2_ (12–14).

The current study found a Spearman correlation coefficient between the O/F and P/F ratios of 0.51, which is lower than that observed for S/F and P/F ratios, generally reported as above 0.61 (10, 21). Calabro et al. (10) found a correlation coefficient between S/F and P/F that was higher than that of O/F; however, the accuracy of S/F was inadequate, as it tended to overestimate P/F. Accuracy was particularly affected when dogs received oxygen supplementation. A retrospective study investigated the effect of oxygen supplementation on the correlation between P/F and SaO_2_/FiO_2_ and reported that providing a mixture of air and oxygen to achieve an FiO_2_ of 0.37 could lead to an underestimation of the P/F ratio by up to 50% (12). The detrimental effect of hyperoxemia on the relevance of the S/F ratio can be mitigated by using the O/F index, as the manufacturer reported that ORi can quantify PaO_2_ levels between 100 and 200 mmHg. Nevertheless, the mild correlation between the O/F and P/F ratios could represent the mild to moderate correlation between PaO_2_ and ORi, as reported by studies in both human and veterinary patients (13, 16).

The anesthetic protocols, particularly the use of alpha-2 agonist sedatives like dexmedetomidine, may affect local perfusion and introduce variability in the accuracy of ORi for estimating PaO_2_. Similarly, the reliability and accuracy of conventional pulse oximetry readings depend on the quality of arterial blood flow at the probe application site. In healthy dogs under general anesthesia, two studies reported mean PI measurements at the tongue ranging from 0.3 to 1.9, with variability induced by tongue width and ambient light exposure (22, 23). In these studies, the animals received an FiO₂ of 1, and although PaO₂ was not measured, SpO₂ was considered consistent. A recent report suggests that PI values greater than 2 can significantly decrease the accuracy of ORi in estimating PaO₂ (19). As vasodilation affects SpO₂ accuracy and elevates PI (24, 25), it may also have a similar effect on ORi, although no studies are available to confirm the hypothesis.

To enhance its clinical applicability, a cut-off for the O/F ratio was identified to help guide ventilation strategies. A ratio above 1.2, with 80% sensitivity, suggests a venous admixture of less than 10%. However, the Youden index from this study indicates that the ratio lacks specificity in detecting dogs with an F-shunt greater than 10%. Clinically, this index could be used to adjust ventilation and FiO_2_, targeting a ratio above this threshold to potentially reduce the negative impact of venous admixture and improve oxygenation during recovery, particularly when the risk of hypoxemia developing rapidly is high (26). Additionally, the index can guide the use of alveolar recruitment maneuvers to decrease the venous admixture and prevent a decline in oxygenation once the patient is removed from the oxygen source (27, 28).

The O/F ratio could be a valuable index for identifying abnormal venous admixture before clinically significant hypoxemia develops. However, while it holds potential diagnostic value, using it solely as a surrogate for the P/F ratio has limitations. The P/F ratio is traditionally used to classify the severity of hypoxemia, regardless of FiO_2_, and a PaO_2_ below 60 mmHg can result from various conditions, including ventilation-perfusion mismatch, anatomic shunt, diffusion impairment, or hypoventilation. Since the O/F ratio is calculated with an FiO_2_ that yields an ORi value greater than 0, corresponding to a PaO_2_ above 100 mmHg, abnormal values in this index cannot accurately quantify the degree of hypoventilation, as it does not account for the impact of PaCO_2_ on oxygenation (29). Additionally, at high FiO_2_ levels, the impact of PaCO_2_ on alveolar oxygen content is minimal, making this index less accurate for classifying hypoventilation. The O/F ratio might be more suitable for assessing pulmonary conditions such as atelectasis or abnormal ventilation-perfusion matching, which are particularly common under anesthesia, especially in patients undergoing specific procedures like laparoscopy or in those with conditions like obesity.

This study has some limitations. Firstly, we recorded only a limited number of observations with an F-shunt greater than 10%. Unlike other species, dogs with a normal body condition score under anesthesia or sedation typically exhibit a minimal amount of venous admixture (1). The use of mechanical ventilation in this study, along with the presence of intrinsic equipment PEEP, further minimized the development of a severe shunting fraction (6). Additionally, the number of dogs with a BCS ≥ 8 was limited to less than 25%, reducing the likelihood of observing the elevated fraction of venous admixture that is commonly seen in this class of patients (7). This study used data from a previous study and added seven new cases to replace paired measurements removed due to PI >2 or ORi values of 0 or 1. Although a sample size calculation was not conducted *a priori*, a retrospective power analysis indicated that the data had a power of 0.96, assuming a Spearman correlation coefficient of 0.59 and a sample size of 77 paired observations. Another limitation of this study is the use of F-shunt as an approximation of venous admixture, which is typically measured invasively from mixed venous blood in the pulmonary artery. While some species demonstrate a high coefficient of determination between these two parameters, variability exists among species (2, 3), and no studies have evaluated the agreement between these variables in dogs.

Another confounding factor that may explain the low ability of the O/F ratio to identify animals with an F-shunt greater than 10% is the non-linear relationship between the P/F ratio and FiO_2_, particularly in cases of high venous admixture, where this ratio increases at both low and high FiO_2_ levels (30). The dogs in this study received a mixture of gases containing varying oxygen fractions, up to 0.66. A study using a mathematical model identified a variability of up to 100 mmHg in the P/F ratio when FiO_2_ ranged from 0.21 to 0.6. This variability also affects SaO_2_, and in cases where ORi is used, there may be further impact since the index also considers venous hemoglobin oxygen saturation (31). The potential effect of different FiO_2_ levels on the O/F ratio was not investigated in this study, and to the authors’ knowledge, no studies in humans have addressed this issue.

## 5 Conclusion

The study demonstrated that the ORi/FiO_2_ ratio could be a non-invasive alternative to the PaO_2_/FiO_2_ ratio for estimating venous admixture in dogs undergoing general anesthesia in a condition of mild hyperoxia. For PI values below 2, an ORi/FiO_2_ value above 1.2 predicted an F-shunt of ≤10% with good sensitivity. However, some dogs with an ORi/FiO_2_ ratio below 1.2 still exhibited an F-shunt ≤10%. This suggests that while the ORi/FiO_2_ ratio is promising, it requires further investigation and does not replace blood gas analysis.

## Data Availability

The raw data supporting the conclusions of this article will be made available by the authors, without undue reservation.
